# *Paecilomyces variotii* Mycoprotein in Diets for Juvenile Rainbow Trout: Effects on Growth Performance, Intestinal Health and Technical Feed Quality

**DOI:** 10.1155/anu/4320030

**Published:** 2026-06-15

**Authors:** Ashwath Gaudhaman, Aleksandar Vidakovic, Niklas Warwas, Darragh Doyle, Byron Morales-Lange, Margareth Øverland, Torbjörn Lundh, Kartik Baruah

**Affiliations:** ^1^ Department of Applied Animal Science and Welfare, Faculty of Veterinary Medicine and Animal Sciences, Swedish University of Agricultural Sciences, Uppsala, Sweden, slu.se; ^2^ Department of Biological and Environmental Sciences, University of Gothenburg, Gothenburg, Sweden, gu.se; ^3^ Department of Animal and Aquacultural Sciences, Faculty of Biosciences, Norwegian University of Life Sciences, Ås, Norway, nmbu.no

**Keywords:** feed utilisation, growth parameters, gut histology, *Oncorhynchus mykiss*, *Paecilomyces variotii*, transepithelial potential

## Abstract

Aquaculture continues to search for new protein sources that can promote fish growth and health, as well as reduce competition between food and feed. Thus, the current study evaluated the dietary inclusion of a *Paecilomyces variotii*‐based microbial ingredient on technical pellet quality, growth performance and intestinal health of juvenile rainbow trout (*Oncorhynchus mykiss*). Five experimental diets were formulated to include 0% (control), 5%, 10%, 20%, and 30% of *P. variotii*. Fish (initial body weight: 43 ± 10 g) were fed with the experimental diets for 9 weeks in triplicate tanks per treatment. Increasing inclusion levels of *P. variotii* significantly influenced feed pellet physical characteristics, with reduced pellet durability but an increased water stability index. Growth performance did not differ significantly between the control group and fish fed diets containing up to 20% *P. variotii*. However, fish fed the 30% *P. variotii* exhibited reduced feed intake and growth, suggesting an upper limit for its dietary inclusion. Moreover, the transepithelial potential (TEP) in the proximal intestine was significantly lower in fish fed the 30% *P. variotii* diet compared with the control, indicating compromised epithelial integrity at higher inclusion levels. Although the villi length was significantly higher at a 30% inclusion level, no significant difference was observed in the goblet cell area among treatments. Overall, under the present diet formulation and processing conditions, the inclusion of *P. variotii* appears feasible at inclusion levels up to 20% (without adverse effects on growth performance or intestinal health). This supports its potential as a sustainable feed ingredient, yet further studies with longer feeding periods and different developmental stages of fish are needed to fully assess its applicability in commercial aquaculture settings.

## 1. Introduction

In recent decades, the rapid expansion of aquaculture has increased the demand for sustainable and nutritionally rich feed ingredients, particularly alternative protein sources that can reduce reliance on fishmeal and mitigate pressure on wild fish stocks [1, 2]. Diversifying protein sources in fish feed is therefore essential to improve environmental sustainability and strengthen feed security in the face of supply chain disruptions driven by environmental and geopolitical challenges [3]. These issues are especially relevant for rainbow trout (*Oncorhynchus mykiss*), a globally important aquaculture species with high dietary protein requirements of ~38% digestible protein [4].

Plant‐based protein sources (e.g., soybean meal and sunflower meal) offer potential alternatives to fishmeal, as they are both inexpensive and abundant. However, their use in salmonid diets may be limited by reduced digestibility, imbalanced amino acid profiles, and the presence of anti‐nutritional factors, which can impair nutrient digestibility, growth performance and gut health [5, 6]. These ingredients also present several environmental challenges, including increased pressure on natural resources, agricultural land, and a higher overall ecological footprint. These limitations have stimulated interest in alternative protein sources that are both nutritionally efficient and environmentally sustainable.

Microbial proteins have emerged as promising candidates for aquafeeds. Multicellular fungi are particularly attractive due to their high protein content, balanced amino acid profiles and the ability to grow on diverse substrates, including industrial and agricultural side streams [7, 8]. This capacity enables the valorisation of organic waste streams while reducing reliance on arable land for protein production [8]. Fungal biomass also generally exhibits a lower carbon footprint compared with that of conventional protein sources used in aquafeeds [9, 10]. Among fungal protein sources, *Paecilomyces variotii* has attracted interest due to its high crude protein content (~67%) and favourable amino acid composition. This filamentous multicellular fungus can be mechanically separated from fermentation media, which simplifies downstream processing and may reduce production costs. Earlier studies in the 1980 s evaluated *P. variotii* as a feed ingredient for terrestrial animals [11, 12], while more recent research has investigated its potential in aquafeeds. In Atlantic salmon (*Salmo salar*), *P. variotii* strain KCL‐24 was shown to replace up to 20% of dietary protein without compromising growth performance [13, 14]. In those studies, the experimental diets were formulated to be isonitrogenous and isoenergetic. However, studies evaluating its application in other salmonid species remain scarce, and the effects of inclusion levels exceeding 20% protein replacement on fish growth performance, digestive function and health status remain unclear.

The digestive tract plays a central role in nutrient digestion, absorption and immune defence in fish [15]. Microbial ingredients, such as filamentous fungi, contain microbe‐associated molecular patterns (MAMPs), such as β‐glucans, mannans, lipoteichoic acids and nucleic acids [14, 16]. These MAMPs can interact with pattern recognition receptors (PRRs) in the gut‐associated lymphoid tissue (GALT), potentially modulating local immune responses and contributing to gut homeostasis [16, 17]. Consequently, the evaluation of intestinal morphology and physiological function provides important insights into the health implications of novel feed ingredients. Histological analyses of intestinal structures, such as villus length, goblet cell number and intestinal permeability, are widely used indicators of gut health and early inflammatory responses in fish [18–21]. In addition to nutritional and physiological considerations, the technical quality of feed pellets is a critical factor when evaluating novel aquafeed ingredients. Pellet characteristics, such as expansion, pellet width, water stability, and sinking velocity, can influence feed performance and waste generation in aquaculture systems [22]. Microbial ingredients have previously been shown to affect these physical pellet properties during feed processing [13].

Based on the nutritional characteristics of fungal proteins and previous findings in Atlantic salmon, we hypothesised that dietary inclusion of *P. variotii* can partially replace conventional protein sources in diets for juvenile rainbow trout without compromising growth performance or intestinal health, while potentially influencing feed technical quality and nutrient utilisation. The objective of this study was therefore to evaluate the impacts of graded inclusion levels of *P. variotii* in diets for rainbow trout on technical pellet quality, nutrient digestibility, growth performance, intestinal morphology and electrophysiology. By integrating nutritional, physiological and feed‐processing perspectives, this study seeks to define practical inclusion limits and assess the feasibility of *P. variotii* as a sustainable protein source for rainbow trout farming.

## 2. Materials and Methods

### 2.1. Microbial Ingredient Production, Experimental Diets and Pellet Quality

The microbial protein used in this study was based on *P. variotii* KCL‐24, which was produced by Enifer (Oy, Espoo, Finland). *P. variotii* was produced using sugar beet molasses, a by‐product of bioethanol production. Briefly, the medium containing per 1000 L of suitably diluted vinasse providing 20 g L^−1^ utilisable carbon sources (mainly glycerol and residual sugars), (NH_4_)_2_SO_4_ 5 kg, KCl 150 g, MgSO_4_ 7 H_2_O 150 g and Vogel’s trace elements was continuously fed at a dilution rate ~0.3 h^−1^ to the aerobic fermentation at 37°C. The microbial material was continuously collected at the same rate and harvested by mechanical filtration using a Larox filter press (Lappeenranta, Finland).

Thereafter, five experimental diets were formulated, consisting of one commercial‐like diet (control) and four test diets containing increasing inclusion levels of *P. variotii* (Table 1). The control diet was formulated to reflect standard industry practice, while the test diets included 5% (*D*_5_), 10% (*D*_10_), 20% (*D*_20_) and 30% (*D*_30_) of *P. variotii*. The inclusion of the microbial ingredient was achieved by proportional replacement of fish meal and soy protein concentrate in the diets. Moreover, yttrium oxide (Y_2_O_3_) was used as an inert marker for nutrient digestibility analysis. All experimental diets were manufactured at the Feed Technology Laboratory at the Swedish University of Agricultural Sciences (SLU) in Uppsala, Sweden, on a Ketse 20/40 twin‐screw extruder (Brabender GmbH & Co. KG, Duisburg, Germany) fitted with a 2 mm die head for pellet production. Then, pellets were dried in a vertical drying oven (Elvärmedetaljer, Skurup, Sweden) and later coated with oil using a mini GVC‐10 vacuum coater (Amandus Kahl GmbH & Co. KG, Reinbek, Germany).

**Table 1:** Dietary composition.

Ingredients (g kg^−1^)	*D*_0_	*D*_5_	*D*_10_	*D*_20_	*D*_30_
Fish meal^a^	390	365	340	290	240
Soy protein concentrate^b^	148	123	100	50	0
Wheat gluten^c^	60	60	60	60	60
Wheat meal^d^	120	120	120	120	120
Potato starch^e^	93	93	88	85.5	80
Fish oil^f^	79	79	80	81	84
Rapeseed oil^g^	80	80	80	80	80
Vitamin mineral premix^h^	10	10	10	10	10
Pekilo	0	50	100	200	300
Lysine sulfate^i^	0	0	1	2	3.7
Choline chloride^j^	5	5	5	5	5
DL‐methionine^k^	0	0.3	0.7	1.6	2.2
Monocalcium phosphate^l^	15	15	15	15	15
Yttrium oxide^m^	0.1	0.1	0.1	0.1	0.1

*Note: D*_0_: control (0% *P. variotii*), *D*_5_: 5% *P. variotii* inclusion, *D*_10_: 10% *P. variotii* inclusion, *D*_20_: 20% *P. variotii* inclusion and *D*_30_: 30% *P. variotii* inclusion.

^a^Group 1 fishmeal, Pelagia, Bergen, Norway.

^b^HP310, Hamlet Protein A/S, Horsens, Denmark.

^c^Repal GL21, Lantmännen Reppe AB, Lidköping, Sweden.

^d^Wheatmeal standard, Axfood AB, Sweden.

^e^Potatismjöl, Axfood AB, Sweden.

^f^Fish oil herring, AB Salmonfarm Oy, Kasnäs, Finland.

^g^Rapeseed oil feed grade, Avena Nordic Grain Oy, Helsinki.

^h^Per kg of premix: Vit A 2,266,667 IU kg^−1^, Vit D3 1,000,000 IU kg^−1^, menadione 6667 mg kg^−1^, thiamine 6000 mg kg^−1^, riboflavin 8667 mg kg^−1^, pantothenic acid 26,667 mg kg^−1^, pyridoxine 5667 mg kg^−1^, Vit B12 20,000 μg kg^−1^, nicotinic acid 50,000 mg kg^−1^, folic acid 3333 mg kg^−1^, biotin 263,667 μg IU kg^−1^, Vit C 90,000 mg kg^−1^, inositol 165,000 mg kg^−1^, zinc 25,000 mg kg^−1^, iodine 1067 mg kg^−1^, copper 1318 mg kg^−1^, manganese 1640 mg kg^−1^, citric acid 180 mg kg^−1^, BHT 536 mg kg^−1^ and BHA 256 mg kg^−1^.

^i^L‐lysine monohydrochloride (L‐Lysine HCl), MEIHUA, Langfang, Hebei, China.

^j^MIAVIT GmbH, Essen, Germany.

^k^MetAMINO DL‐methionine, Evonik Nutrition & Care GmbH, Essen, Germany.

^l^MCP—Monocalcium phosphate, Aako, Leusden, Netherlands.

^m^Yttrium oxide (Y_2_O_3_) Sigma–Aldrich Sweden AB, Stockholm, Sweden.

To analyse the quality of the pellet, 30 finished oil‐coated pellets of each diet were randomly selected and arranged based on their length in ascending order. The middle 15 pellets were chosen for analysis, and the width and hardness were measured on the same pellets. First, the length and width of the 15 pellets were measured using electronic callipers. Then, the hardness (the force needed to break the pellets, measured in kg) was measured using a hand‐held hardness tester (Herkules M, Amandus Kahl GmbH & Co. KG, 21,465 Reinbek, Germany). The pellet durability was measured using a NHP100 (New Holmen portable pellet tester, Holmen Feed, Norfolk, UK) according to Wolska et al. [23]. Briefly, pellets were pre‐sieved to remove any possible fines and crumbles. After that, 100 g of pellets was then subjected to 70 mbar pressure for 120 s while being air/tumbled continuously. The remaining pellets were subsequently weighed to calculate the pellet durability index. The test was done in triplicate for each diet, and the apparatus was cleaned to remove oil and debris between each time. Regarding sinking velocity (m s^−1^), this was calculated by dropping randomly selected 35 pellets into a still‐water‐filled transparent tube and measuring the time it took for the pellets to sink 1 m at 20°C. Water stability was calculated following the method described by Baeverfjord et al. [24], with modified incubation times of 30, 90 and 180 min.

### 2.2. Fish Experiment

The experimental protocol involving fish was approved by the Institutional Ethical Committee Board of the Swedish Board of Agriculture (5.8.18–23275/2022). The study was conducted at the Aquatic Research Facility of the Faculty of Veterinary Medicine and Animal Science, SLU, Uppsala. Juvenile rainbow trout (450), with an average initial weight of ~43 ± 10 g, were obtained from Vilstena fiskodling. Fish were randomly distributed into tanks (30 individuals per tank), ensuring similar initial mean body weight across all tanks to minimise variation. A total of 15 experimental tanks were used for the trial, each with a capacity of 200 L. Fish were reared under control conditions with a 12:12‐h light cycle, a water temperature of 12.9 ± 0.3°C, and a DO level of 8.8 ± 0.3 mg L^−1^. Fish were fed to satiation twice daily at 11:00 and 15:00 by automated belt feeders (Hølland teknologi, Sandnes, Norway), with each feeding session lasting for an hour. The faeces from the previous day were collected daily just before the start of each feeding period, and weighed periodically. The uneaten feed was separated from faeces, and both were stored at −20°C. Feeding rations were determined based on the amount of uneaten feed recovered from the previous day. The fish were individually weighed at the start and end of the experiment.

(MS‐222; Western Chemical Inc., Ferndale, WA, USA) at 40 mg L^−1^, and their weight and length were recorded. Six fish per tank were randomly sampled for histology, Ussing chamber analysis, hepatosomatic index (HSI), and viscerosomatic index (VSI). For this, the fish were euthanized with an MS‐222 overdose (200 mg L^−1^) prior to a quick blow to the head. Following euthanasia, their viscera were removed and weighed. Afterwards, their livers were removed and weighed. Then, the fourth lobe of the liver was collected and immediately frozen in liquid nitrogen using cryotubes. The head kidney was removed by making an incision to mark the organ boundary at the narrowest point and by scraping the tissue using a blunt tool. The muscle tissue was sampled from ~2.5 cm posterior to the dorsal fin down to the lateral line. The skin was removed, and the muscle tissue underneath was taken in cryotubes and snap‐frozen in liquid nitrogen. Also, the spleen was cut from the viscera, transferred to cryotubes and snap‐frozen in liquid nitrogen. The distal intestine was dissected at the ileorectal valve and ~1 mm from the anus. The intestinal segment was cut open, and the contents were gently rinsed using PBS. A 1–2 cm section from the proximal part of the distal intestine was folded inside out and fixed in 10% phosphate‐buffered formalin for histological analysis. The remaining distal part was collected in cryotubes and snap‐frozen in liquid nitrogen.

### 2.3. Proximate Composition Analysis

*P. variotii* samples, milled feed samples and faecal samples (freeze‐dried and subsequently milled) were used to determine their proximate composition and amino acid profile. The dry matter content was determined by oven‐drying subsamples at 103°C for 16 h. Following drying, samples were cooled in a desiccator and weighed. Ash content was measured by incinerating the dried samples in a muffle furnace at 550°C for 3 h, after which the samples were cooled in a desiccator and then weighed. The total nitrogen content was quantified using the Kjeldahl method, employing a 20‐digester, an 8400 Kjeltec analyser and an 8460‐sampler unit (Foss, Sweden). The crude protein content was calculated by multiplying the nitrogen content by a factor of 6.25, whereas the crude fat content was assessed using the Soxhlet extraction method [25]. Yttrium levels were measured spectrophotometrically using a Microwave Plasma Atomic Emission Spectrometer (MP‐AES‐4200, Santa Clara, CA, USA). In addition, amino acid profiling was carried out using a liquid chromatography coupled with tandem mass spectrometry (HPLC) method by Eurofins Biopharma Product Testing Sweden AB (Uppsala, Sweden). The proximate composition and amino acid profile of the dried *P. variotii* biomass and the experimental diets are summarised in Tables 2 and 3, respectively.

**Table 2:** Proximate analysis of *P. variotii* mycoprotein on a dry matter (DM) basis.

Proximate composition	*P. variotii*
Dry matter (%)	94.2
Ash content (g kg^−1^ of DM)	93
Crude protein (g kg^−1^ of DM)	668
Crude fat (g kg^−1^ of DM)	41
Gross energy (MJ kg^−1^)	21.3
Essential amino acids (g kg^−1^ of DM)	
Arginine	34.6
Histidine	11.2
Isoleucine	21.6
Leucine	38.9
Lysine	38.0
Methionine	8.4
Phenylalanine	20.7
Threonine	24.4
Valine	25.9
Non‐essential amino acids (g kg^−1^ of DM)	
Alanine	37.3
Aspartic acid	46.9
Cystein + cystine	5.4
Glutamic acid	62.9
Glycine	26.3
Proline	25.8
Serine	25.0

**Table 3:** Proximate composition of diets.

Ingredients	*D*_0_	*D*_5_	*D*_10_	*D*_20_	*D*_30_
Dry matter (%)	94.2	94.5	94.1	94.4	94.7
Ash content (g kg^−1^ of DM)	105	104	108	98	99
Crude protein (g kg^−1^ of DM)	470	471	483	474	531
Crude fat (g kg^−1^ of DM)	222	236	181	190	133
Gross energy (MJ kg^−1^)	22.0	21.7	21.4	21.7	20.3
Essential amino acids (g kg^−1^ DM)					
Arginine	26	25	25	26	26
Histidine	9	9	9	9	9
Isoleucine	17	17	18	17	18
Leucine	32	32	32	31	32
Lysine	28	28	28	29	30
Methionine	9	10	10	11	12
Phenylalanine	19	19	20	19	19
Threonine	17	17	17	17	18
Valine	19	19	20	20	21
Non‐essential amino acids (g kg^−1^ DM)					
Alanine	22	22	23	24	25
Aspartic acid	40	39	40	38	37
Cystine	6	6	6	5	5
Glutamic acid	81	79	80	77	75
Glycine	24	24	24	24	24
Proline	25	25	25	26	26
Serine	20	20	21	20	20

*Note: D*_0_: control (0% *P. variotii*), *D*_5_: 5% *P. variotii* inclusion, *D*_10_: 10% *P. variotii* inclusion, *D*_20_: 20% *P. variotii* inclusion and *D*_30_: 30% *P. variotii* inclusion.

Abbreviation: DM, dry matter.

### 2.4. Histological Analysis

**Figure 1:**
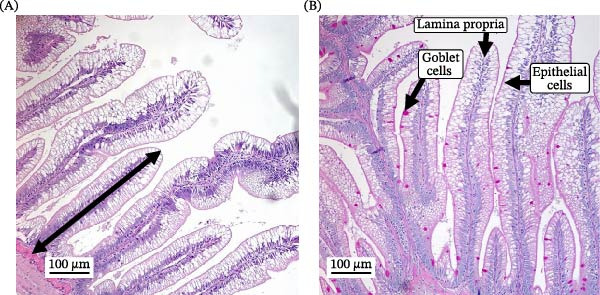
Representative histological measurement. (A) Villi length (indicated by black, double‐headed arrow). (B) Goblet cell and other morphometric regions.

The distal intestine samples, ~3–5 mm in length, were fixed in 10% formalin for 48 h. The sample was then dehydrated with increasing concentrations (50%–100%) of ethanol, as described by Purushothaman et al. [26]. The dehydrated samples were embedded in paraffin and were sectioned in 5 µm cross sections and stained with periodic acid‐Schiff (PAS) and haematoxylin and eosin (HE) following the protocol described by Hellman et al. [27]. The stained tissue samples were captured using a Nikon microscope and a DXM1200 digital camera operated through ACT‐1 software (v.2.70). Villi length and goblet cell area were calculated using ImageJ (v.1.54g) as described by Rocha et al. [28] and Raskovic et al. [29]. Representative histological measurements (i.e., villi length, goblet cell and other morphometric regions) are presented in Figure 1.

### 2.5. Ussing Chamber Analysis

To evaluate the effect of *P. variotii* on intestinal permeability and ion transport, ex vivo measurements were performed using the Ussing chamber technique as described by Warwas et al. [30]. Briefly, the intestine was divided into proximal (from the final pyloric caecum to the ileorectal valve) and distal (from the ileorectal valve to the anus) regions. Both intestinal sections were opened longitudinally and mounted in modified Ussing chambers. Four millilitres of chilled Ringer’s solution were added to each side of the chamber, which was continuously aerated with air. The temperature was maintained at 12°C using a water‐cooled mantle. Electrical measurements were taken as follows: transepithelial potential (TEP) difference was continuously measured using two Ag/Cl electrodes. Every 5 min, alternating DC voltages (U) were applied across the epithelium using two nitinol electrodes, which generated corresponding currents (I; ranging from −30 to 30 μA). A line was fitted to the U I^−1^ pairs using the least‐squares method. The slope of this line represented the transepithelial resistance (TER). Where the line intersected the *U* axis at 0 was the short‐circuit current (SCC). In biological tissues, TER serves as a proxy measurement of epithelial permeability or integrity. In the intestine of fish, the TER is generally a measure of the paracellular shunt resistance of the epithelial layer. TEP reflects the net distribution of charged particles between the two chamber compartments and is an indicator of the passive and active transport processes occurring across the epithelium. The SCC represents the net flow of charged particles across the epithelium and provides a direct estimate of active electrogenic transport. After 60 min of acclimation, Ringer’s solution was renewed to ensure identical conditions on both sides of the epithelia. The experimental period was 90 min, starting from the end of acclimation.

### 2.6. Growth Performance, Feed Utilisation and Pellet Quality Calculations

Specific growth rate SGR=Ln final body weight; FBW−Ln initial body weight; IBWexperimental period days×100,

Thermal unit growth coefficient=100×FBW13−IBW13∑T×D,

Expansion %=pellet width−die diameterdie diameter×100,

Growth rate %=FBW−IBWIBW×100,

Mortality adjusted initial weight g=total initial weight g−average initial weight g×number of mortality,

Corrected weight gain g =final weight g−mortality adjusted initial weight g,

Corrected FCR=feed intake gDM/fishcorrected weight gain g ,

Nutrient retention=FBW×nutrient contentfinal−IBW×nutrient contentinitial,

Nutrient retention efficiency %=FBW× nutrient contentfinal−IBW×nutrient contentinitial% ingested or % digested×100,

Protein production value=FBW×protein contentfinal−IBW×protein contentinitialfeed intake g/fish×protein content in the feed %,

ADCdry matter =1−markerfeed/markerfaeces,

AD %=ADC×100,

ADCnutrient/energy=1−markerfeed×nutrientfaecesmarkerfaeces×nutrientfeed,

Markerfeed=marker content as % of dry matter of the feed,

Markerfaeces=marker content as % of dry matter of the faeces,

Nutrientfeed=nutrient content as % of dry matter of the feed,

Nutrientfaeces=nutrient content as % of dry matter of the faeces.

### 2.7. Statistical Analysis

All statistical analyses were performed using RStudio (v.2024.04.2, Build 764). Data were tested for normality and homogeneity of variance as criteria for analysis of variance (ANOVA). Normality was tested using the Shapiro–Wilk method and Q–Q plots. Homogeneity of variance was tested using the Levene’s test. One‐way ANOVA was performed with the dietary inclusion level as the fixed factor. Differences were considered significant at *p* < 0.05. When the results were significant, estimated marginal means (EMMEANS) were used for post hoc comparisons among different groups to identify specific significant differences. Linear and quadratic regression analyses were performed with dietary inclusion levels as continuous variables, and the corresponding *p*‐values are reported as *p*‐linear and *p*‐quadratic. In addition, the Akaike information criterion (AIC) was calculated for linear and quadratic models when *p* was <0.05.

## 3. Results

### 3.1. Extruder Parameters and Pellet Quality

The extruder parameters for the different diets (*D*_0_–*D*_30_) are summarised in Table 4. The control diet (*D*_0_) was processed at the lowest cutter speed of 600 RPM, whereas higher cutter speeds of 690 RPM were used for the *D*_5_ and *D*_10_ diets. Diets *D*_20_ and *D*_30_ were prepared at an intermediate cutter speed of 660 RPM. The pressure at the die increased progressively with diet level, rising from 18.1 bar in *D*_0_ to 28.3 bar in the *D*_30_ diet. The screw speed was maintained at 230 RPM for control, *D*_5_ and *D*_10_ but was increased to 280 RPM for *D*_20_ and *D*_30_. The water addition was constant at 0.04 L min^−1^ for the *D*_0_ to *D*_20_ diets, but it was slightly reduced to 0.03 L min^−1^ for *D*_30_. The feeding rate was 4.5 kg h^−1^ for *D*_0_, *D*_5_ and *D*_10_ and 4.2 kg h^−1^ for *D*_20_ and *D*_30_ diets. The extruder load varied from 18% in *D*_0_ to 25% in *D*_30_, indicating a higher mechanical demand for processing diets with higher inclusion of microbial ingredients. The barrel temperature profiles also differed between diets. The temperatures in zones 1–4 were similar in all diets except for the control (*D*_0_). Increased expansion in experimental diets necessitated adjustments to temperatures in heating zones one to five, as well as screw speed, to ensure consistent pellet density and oil addition.

**Table 4:** Extruder parameters when producing the experimental diets containing increasing levels of *P. variotii*.

Diets	Cutter (RPM)	Die pressure (bar)	Screw (RPM)	Water addition (L min^−1^)	Feeding rate (kg h^−1^)	Load (%)	Barrel temperature (°C)				
							Z1	Z2	Z3	Z4	Z5
*D*_0_	600	18.1	230	0.04	4.5	18	110	130	130	110	100
*D*_5_	690	20.8	230	0.04	4.5	20	80	90	110	105	100
*D*_10_	690	21.7	230	0.04	4.5	20	80	90	110	105	100
*D*_20_	660	23.6	280	0.04	4.2	19	80	90	105	105	100
*D*_30_	660	28.3	280	0.03	4.2	25	80	90	105	105	100

*Note: D*_0_: control (0% *P. variotii*), *D*_5_: 5% *P. variotii* inclusion, *D*_10_: 10% *P. variotii* inclusion, *D*_20_: 20% *P. variotii* inclusion and *D*_30_: 30% *P. variotii* inclusion.

Abbreviation: Z, zone.

The physical pellet quality parameters for diets *D*_0_–*D*_30_ are presented in Table 5. The pellet width and the pellet expansion did not vary significantly among diets (*p* > 0.05). The pellet durability was significantly influenced by dietary inclusion of the test ingredients (*p* < 0.0001). Diets *D*_0_, *D*_5_, *D*_10_ and *D*_20_ exhibited similar high durability values (89.2%–91.1%). However, the *D*_30_ had a significant reduction in durability value (71.5%). The durability exhibited a quadratic decrease with increasing inclusion levels (*p*_value_ < 0.0001, *p*_linear_ = 0.0019 and *p*_quadratic_ < 0.0001). Sinking velocity differed significantly among diets (*p* = 0.002). Pellets from *D*_5_ exhibited the lowest sinking velocity (0.060 m s^−1^), while *D*_0_ showed the highest value (0.072 m s^−1^), and it was not significantly different from that of the *D*_10_, *D*_20_ and *D*_30_ diets. There was no linear or quadratic relationship between the diets and the sinking velocity. A linear relationship was observed, with water stability after 30 min increasing as the inclusion levels of *P. variotii* increased (*p*_value_ = 0.0292, *p*_linear_ = 0.0005 and *p*_quadratic_ = 0.0028). However, at 90 and 180 min, a significant quadratic relationship was observed (*p*_value_ = 0.0289, *p*_linear_ = 0.0045 and *p*_quadratic_ = 0.0092). The water stability remained the highest for diet *D*_30_ at all the tested time points.

**Table 5:** Physical pellet quality along with the water stability index of the different diets.

Parameters	*D*_0_	*D*_5_	*D*_10_	*D*_20_	*D*_30_	SEM	*p*_value_	*p*_linear_	*p*_quadratic_	AIC
Pellet width (mm)	2.53	2.64	2.48	2.63	2.53	0.05	0.0756	NA	NA	NA
Expansion (%)	26.50	32.13	24.00	31.63	26.50	0.27	0.0756	NA	NA	NA
Durability (%)	90.6^a^	91.1^a^	89.2^a^	90.5^a^	71.5^b^	0.46	<0.0001	0.0019	<0.0001	Q
Sinking velocity (m sec^−1^)	0.072^a^	0.0600^b^	0.0679^ab^	0.0668^ab^	0.0679^ab^	0.0021	0.002	0.806	0.097	Q
Water stability index (%)										
30 min	88^b^	89^ab^	90^ab^	91^ab^	92^a^	0.01	0.0292	0.0005	0.0028	L
90 min	83^b^	85^b^	85^b^	86^ab^	89^a^	0.01	0.003	0.0002	0.0006	Q
180 min	78^ab^	76^b^	80^ab^	80^ab^	83^a^	0.01	0.0289	0.0045	0.0092	Q

*Note:* Different superscript letters within a row indicate statistically significant differences between means (*p* < 0.05). In addition, *p*_value_ refers to the ANOVA, whereas *p*_linear_ refers to the linear regression model and *p*_quadratic_ refers to the quadratic regression model. When *p* was <0.05, the Akaike information criterion (AIC) was used to determine the model that best describes the distribution (L: linear or Q: quadratic). When *p* was >0.05, AIC was not analysed (NA). *D*_0_: control (0% *P. variotii*), *D*_5_: 5% *P. variotii* inclusion, *D*_10_: 10% *P. variotii* inclusion, *D*_20_: 20% *P. variotii* inclusion and *D*_30_: 30% *P. variotii* inclusion.

### 3.2. Growth Performance

Growth parameters and body indices are shown in Table 6. The initial weight of the fish did not differ significantly between the different experimental groups. The final body weight was significantly lower in fish fed *D*_30_ compared with all other diets. There was also a negative and quadratic relationship between inclusion levels and final weight (*p*_value_ = 0.0062, *p*_linear_ = 0.0219 and *p*_quadratic_ = 0.0019). Weight gain (%) followed a similar pattern, with *D*_30_ resulting in significantly lower values compared with those of the control, *D*_10_ and *D*_20_ diets. A significant negative linear and quadratic relationship was observed between the inclusion levels of *P. variotii* and weight gain (*p*_value_ = 0.0127, *p*_linear_ = 0.0149, and *p*_quadratic_ = 0.0037). The specific growth rate was also significantly lower in *D*_30_ compared with that in the other inclusion levels. A significant negative linear correlation was observed between the different inclusion levels and SGR (*p*_value_ = 0.0076, *p*_linear_ = 0.0315 and *p*_quadratic_ = 0.0071). The feed intake was significantly lower for *D*_30_ compared to all diets except *D*_5,_ and a quadratic relationship was observed between increasing levels of *P. variotii* and feed intake (*p*_value_ = 0.0127, *p*_linear_ = 0.0149 and *p*_quadratic_ = 0.0037). In contrast, the corrected FCR was not significantly different between the different groups. There was a significant positive linear correlation between the inclusion levels and VSI (*p*_value_ = 0.0173, *p*_linear_ = 0.0065 and *p*_quadratic_ = 0.0283). The HSI did not differ significantly between the different groups (*p*  > 0.5).

**Table 6:** Growth performance of rainbow trout fed different diets.

Parameters	*D*_0_	*D*_5_	*D*_10_	*D*_20_	*D*_30_	SEM	*p*_value_	*p*_linear_	*p*_quadratic_	AIC
Initial body weight (g)	42.8	43.1	43.5	43.6	42.5	0.30	0.0579	NA	NA	NA
Final body weight (g)	233.2^a^	231 ^a^	238.1^a^	240.6^a^	190.1^b^	7.88	0.0062	0.0219	0.0019	Q
Weight gain (%)	445.7^a^	434.8^ab^	447^a^	451.4^a^	347.1^b^	18.64	0.0127	0.0149	0.0037	Q
SGR (% day^−1^)	2.2^a^	2.2^a^	2.2^a^	2.2^a^	1.9^b^	0.05	0.0076	0.0315	0.1768	L
Feed intake (g tank^−1^)	4115.13^a^	3957.35^ab^	4192.2^a^	4297.71^a^	3201.02^b^	183.72	0.0119	0.0944	0.0246	Q
Corrected FCR	0.72	0.71	0.72	0.73	0.73	0.01	0.6436	NA	NA	NA
VSI	10.70^b^	10.93^ab^	12.07^ab^	11.32^ab^	12.36^a^	0.32	0.0173	0.0065	0.0283	L
HSI	1.21	1.24	1.33	1.33	1.45	0.07	0.2403	NA	NA	NA

*Note:* Different superscript letters within a row indicate statistically significant differences between means (*p* < 0.05). In addition, *p*_value_ refers to the ANOVA, whereas *p*_linear_ refers to the linear regression model and *p*_quadratic_ refers to the quadratic regression model. When *p* was <0.05, the Akaike information criterion (AIC) was used to determine the model that best describes the distribution (L: linear or Q: quadratic). When *p* was >0.05, AIC was not analysed (NA). *D*_0_: control (0% *P. variotii*), *D*_5_: 5% *P. variotii* inclusion, *D*_10_: 10% *P. variotii* inclusion, *D*_20_: 20% *P. variotii* inclusion and *D*_30_: 30% *P. variotii* inclusion.

Abbreviations: HIS, hepatosomatic index; VSI, viscerosomatic index.

### 3.3. Apparent Digestibility (%)

The apparent nutrient digestibility of the diets is presented in Table 7. No significant differences were observed between the inclusion levels and apparent dry matter digestibility % (AD_DM_). The apparent digestibility of ash (AD_Ash_) was significantly influenced by diet (*p*_value_ = 0.0291). Although pairwise comparisons revealed no significant differences between individual dietary groups, AD_Ash_ showed a clear response to increasing inclusion level, with both significant linear (*p* = 0.0013) and quadratic (*p* = 0.0072) trends. For crude protein apparent digestibility % (AD_CP_), no significant differences were found between groups (*p*  > 0.05).

**Table 7:** Apparent digestibility of the different diets.

Parameters	*D*_0_	*D*_5_	*D*_10_	*D*_20_	*D*_30_	SEM	*p*_value_	*p*_linear_	*p*_quadratic_	AIC
Dry matter (%)	84.11	84.2	83.04	84.15	82.04	0.63	0.13	NA	NA	NA
Ash (%)	46.72^a^	47.93^a^	49.71^a^	54.17^a^	53.44^a^	1.60	0.03	0.0013	0.0072	L
Crude protein (%)	94.07	94.24	93.83	93.43	93.32	0.32	0.25	NA	NA	NA

*Note:* Different superscript letters within a row indicate statistically significant differences between means (*p* < 0.05). In addition, *p*_value_ refers to the ANOVA, whereas *p*_linear_ refers to the linear regression model and *p*_quadratic_ refers to the quadratic regression model. When *p* was <0.05, the Akaike information criterion (AIC) was used to determine the model that best describes the distribution (L: linear or Q: quadratic). When *p* was >0.05, AIC was not analysed (NA). *D*_0_: control (0% *P. variotii*), *D*_5_: 5% *P. variotii* inclusion, *D*_10_: 10% *P. variotii* inclusion, *D*_20_: 20% *P. variotii* inclusion and *D*_30_: 30% *P. variotii* inclusion.

### 3.4. Whole‐Body Composition and Nutrient Retention

No significant differences in whole‐body compositions were observed between the different dietary groups (Table 8). However, some differences were observed in nutrient retention (Table 9). Nitrogen retention was significantly lower in fish fed the *D*_30_ diet compared with that in all other diets. There was also a significant negative quadratic correlation with inclusion levels (*p*_value_ = 0.0076, *p*_linear_ = 0.0397 and *p*_quadratic_ = 0.0111). Similarly, energy retention was also significantly lower in fish fed the *D*_30_ diet compared with other groups, with a significant negative quadratic correlation associated with the inclusion levels. The nutrient retention of crude fats was significantly lower for *D*_30_ compared with *D*_20_ levels but not compared with the rest of the diets. There was also a significant negative quadratic correlation observed, *p*_value_ = 0.0324, *p*_linear_ = 0.152 and *p*_quadratic_ = 0.0132. The phosphorus retention levels were significantly lower for *D*_30_ compared to all other diets except *D*_5_. There was also a significant negative quadratic relationship observed with inclusion levels (*p*_value_ = 0.0173, *p*_linear_ = 0.0182 and *p*_quadratic_ = 0.0164). The magnesium, potassium, sodium and sulphur retention levels were significantly lower for *D*_30_ compared with the rest of the diets. There was also a significant negative quadratic relationship with inclusion levels observed for these minerals (*p*_quadratic_ < 0.01).

**Table 8:** Whole‐body composition of the various macro/micro‐nutrients for the fish fed different diets.

Parameters	*D*_0_	*D*_5_	*D*_10_	*D*_20_	*D*_30_	SEM	*p*_value_	*p*_linear_	*p*_quadratic_	AIC
Ash	6.2	5.7	6.01	5.85	5.73	0.12	0.09	NA	NA	NA
Crude protein	49.1	48.4	48.1	48.3	48.7	0.8	0.89	NA	NA	NA
Crude fat	41.1	43.1	42.4	42.49	42.32	0.8	0.51	NA	NA	NA
Gross energy (MJ kg^−1^)	26.0	26.4	26.1	26.5	26.1	0.2	0.40	NA	NA	NA
Calcium (g fish^−1^)	11.5	11.3	10.6	10.51	11.58	0.63	0.62	NA	NA	NA
Potassium (g fish^−1^)	10.0	9.7	9.7	9.8	9.8	0.23	0.66	NA	NA	NA
Magnesium (g fish^−1^)	0.84	0.82	0.82	0.817	0.829	0.015	0.80	NA	NA	NA
Sodium (g fish^−1^)	2.4	2.4	2.38	2.32	2.39	0.06	0.88	NA	NA	NA
Phosphorous (g fish^−1^)	12.1	11.7	11.6	11.51	11.98	0.27	0.66	NA	NA	NA
Sulphur (g fish^−1^)	5.3	5.2	5.2	5.2	5.2	0.09	0.95	NA	NA	NA

*Note:* Different superscript letters within a row indicate statistically significant differences between means (*p* < 0.05). In addition, *p*_value_ refers to the ANOVA, whereas *p*_linear_ refers to the linear regression model and *p*_quadratic_ refers to the quadratic regression model. When *p* was <0.05, the Akaike information criterion (AIC) was used to determine the model that best describes the distribution (L: linear or Q: quadratic). When *p* was >0.05, AIC was not analysed (NA). AIC stands for Akaike information criterion, which is used to decide which model describes the dataset better. *D*_0_: control (0% *P. variotii* inclusion), *D*_5_: 5% *P. variotii* inclusion, *D*_10_: 10% *P. variotii* inclusion, *D*_20_: 20% *P. variotii* inclusion and *D*_30_: 30% *P. variotii* inclusion.

**Table 9:** Nutrient retention of fish fed different experimental diets with increasing levels of *P. variotii*.

Parameters	*D*_0_	*D*_5_	*D*_10_	*D*_20_	*D*_30_	SEM	*p*_value_	*p*_linear_	*p*_quadratic_	AIC
Nitrogen (g fish^−1^)	86.88^a^	83.89^a^	86.87^a^	88.40^a^	66.50^b^	1.54	0.0076	0.0397	0.0111	Q
Energy (kJ fish^−1^)	49.11^a^	49.27^a^	50.59^a^	52.21^a^	38.29^b^	2.15	0.0073	0.0867	0.0095	Q
Crude fat (g fish^−1^)	77.23^a^	80.66^a^	82.27^a^	83.62^a^	62.03^b^	4.35	0.0324	0.152	0.0132	Q
Phosphorous (g fish^−1^)	23.33^a^	21.87^ab^	22.60^ab^	22.66^ab^	17.77^b^	0.99	0.0173	0.0182	0.0164	Q
Magnesium (g fish^−1^)	1.6^a^	1.52^a^	1.59^a^	1.61^a^	1.23^b^	0.05	0.0014	0.0262	0.0064	Q
Potassium (g fish^−1^)	19.26^a^	18.28^a^	19.17^a^	19.41^a^	14.61^b^	0.51	0.0003	0.0201	0.0037	Q
Calcium (g fish^−1^)	22.14	21.06	20.39	20.57	17.32	1.54	0.3142	NA	NA	NA
Sodium (g fish^−1^)	4.53^a^	4.50^a^	4.67^a^	4.57^a^	3.55^b^	0.15	0.0023	0.0251	0.0014	Q
Sulphur (g fish^−1^)	10.15^a^	9.76^a^	10.27^a^	10.31^a^	7.70^b^	0.33	0.0011	0.0295	0.004	Q

*Note:* Different superscript letters within a row indicate statistically significant differences between means (*p* < 0.05). In addition, *p*_value_ refers to the ANOVA, whereas *p*_linear_ refers to the linear regression model and *p*_quadratic_ refers to the quadratic regression model. When *p* was <0.05, the Akaike information criterion (AIC) was used to determine the model that best describes the distribution (L: linear or Q: quadratic). When *p* was >0.05, AIC was not analysed (NA). *D*_0_: control (0% *P. variotii*), *D*_5_: 5% *P. variotii* inclusion, *D*_10_: 10% *P. variotii* inclusion, *D*_20_: 20% *P. variotii* inclusion and *D*_30_: 30% *P. variotii* inclusion.

### 3.5. Histology

The villi length and the goblet cell area in the proximal intestine are presented in Table 10. The villi length was significantly lower in *D*_5_ compared with *D*_30_ and showed a significant positive linear relationship with increasing inclusion levels of *P. variotii* (*p*_value_ = 0.03024, *p*_linear_ = 0.0044 and *p*_quadratic_ = 0.0196). In contrast, the goblet cell area did not vary significantly among the different dietary groups.

**Table 10:** Gut health parameters of fish fed different experimental diets with increasing levels of *P. variotii*.

Parameters	*D*_0_	*D*_5_	*D*_10_	*D*_20_	*D*_30_	SEM	*p*_value_	*p*_linear_	*p*_quadratic_	AIC
Villi length (µm)	857.9^ab^	825.7^b^	904^ab^	934.9^ab^	939.8^a^	23.57	0.0302	0.0044	0.0196	L
Goblet cell area (%)	3.3	3.5	3.6	3.3	3.6	0.3446	0.9104	NA	NA	NA

*Note:* Different superscript letters within a row indicate statistically significant differences between means (*p* < 0.05). *p*_value_ refers to the ANOVA, whereas *p*_linear_ refers to the linear regression model and *p*_quadratic_ refers to the quadratic regression model. When *p* was <0.05, the Akaike information criterion (AIC) was used to determine the model that best describes the distribution (L: linear or Q: quadratic). When *p* was >0.05, AIC was not analysed (NA). The unit % for goblet cell area represents the percentage of the intestinal epithelial area occupied by goblet cells. *D*_0_: control (0% *P. variotii*), *D*_5_: 5% *P. variotii* inclusion, *D*_10_: 10% *P. variotii* inclusion, *D*_20_: 20% *P. variotii* inclusion and *D*_30_: 30% *P. variotii* inclusion.

### 3.6. Ussing Chamber

**Figure 2:**
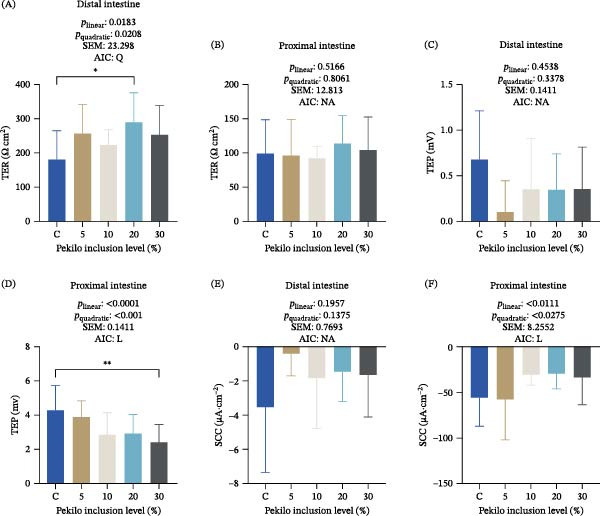
Ussing chamber results for transepithelial resistance (TER) in distal intestine (A) and proximal intestine (B). Transepithelial potential (TEP) of distal intestine (C) and proximal intestine (D). Short‐circuit current (SCC) of distal intestine (E) and proximal intestine (F). The groups that were tested significantly different from each other using ANOVA post hoc were denoted by  ^∗^ for *p* ≤ 0.05 and by  ^∗∗^ for *p* ≤ 0.005. Also, *p*_linear_ and *p*_quadratic_ were used to denote the significance of the regression models. When *p* was ≤ 0.05, the Akaike information criterion (AIC) was used to determine the model that best describes the distribution (L: linear or Q: quadratic). When *p* was >0.05, AIC was not analysed (NA). SEM denotes the standard error over mean for the values. Pekilo: *P. variotii*, C: *D*_0_ (control with 0% *P. variotii* inclusion), 5: *D*_5_ (5% *P. variotii* inclusion), 10: *D*_10_ (10% *P. variotii* inclusion), 20: *D*_20_ (20% *P. variotii* inclusion) and 30: *D*_30_ (30% *P. variotii* inclusion).

As shown in Figure 2A, the TER of the distal intestine was significantly lower in the control group compared with that of *D*_20_ and showed a significant positive quadratic relationship with increasing inclusion levels. Conversely, TER of the proximal intestine was not significantly influenced by diet, and no clear relationship with the inclusion level was observed (Figure 2B). The TEP of the distal intestine did not differ significantly among dietary groups (Figure 2C). In contrast, the TEP of the proximal intestine was significantly lower in *D*_30_ compared with that in the control diet and showed a significant negative linear relationship with increasing inclusion levels (Figure 2D). The SCC of the distal intestine was not significantly affected by diet (Figure 2E). The SCC of the proximal intestine did not differ significantly between the different dietary groups; however, there is a significant positive linear correlation with increasing inclusion levels (*p*_value_ = 0.0587, *p*_linear_ = 0.0111 and *p*_quadratic_ = 0.0275).

## 4. Discussion

*P. variotii* biomass was incorporated into experimental diets at increasing inclusion levels (5%, 10%, 20% and 30%) to systematically assess its effects on feed‐processing characteristics, pellet quality, growth performance, nutrient retention and intestinal health. The use of graded inclusion levels allows for the identification of both linear and non‐linear responses and helps define the threshold at which beneficial effects may transition to potential negative outcomes, thereby supporting the determination of a safe and effective inclusion range for practical rainbow trout feed formulation. The proximate composition of the experimental diets showed that the crude protein level ranged from 470 g kg^−1^ in the control diet (*D*_0_) to 531 g kg^−1^ in *D*_30,_ suggesting that the experimental diets were not isonitrogenous. The crude fat content decreased with increasing dietary inclusion level, from 222 g kg^−1^ in *D*_0_ to 133 g kg^−1^ in *D*_30_. This reduction was reflected in a gradual decline in the gross energy content, which ranged from 22.0 MJ kg^−1^ in *D*_0_ to 20.3 MJ kg^−1^ in *D*_30_. Most of the essential and non‐essential amino acids were comparable across the experimental diets, although lysine and particularly methionine levels showed a relatively higher level in the *D*_30_ diet compared with those in the other groups, especially the control.

Our results also suggested that under the present diet formulation and processing conditions, the inclusion of *P. variotii* up to 20% was well accepted by fish. However, at a higher inclusion level, consistent negative effects were observed across many of the tested biological and technological parameters. For instance, during the feed production, having higher inclusion levels of *P. variotii* required adjustments in extrusion conditions such as water addition and screw speed, resulting in higher die pressure and extruder load, particularly in the *D*_20_ and *D*_30_ diets. These changes suggest increased resistance to flow and possibly altered rheological properties of the feed matrix, which are commonly observed when alternative protein ingredients with different physicochemical characteristics are incorporated into aquafeeds [31, 32]. Such processing‐related differences also influenced the final pellet properties, contributing to deviations from the intended nutritional profiles, especially in relation to oil addition and resulting dietary protein and energy levels.

Data from this study suggested that increasing the inclusion levels of *P. variotii* tended to improve the water stability index but reduce pellet durability. This difference in pellet quality highlights the distinct physicochemical mechanisms governing pellet resistance under dry versus hydrated conditions. Similar dissociations between pellet durability and water stability have been reported in aquafeeds containing alternative or microbial ingredients, indicating that mechanical strength and water stability are not necessarily correlated [22, 33]. Additionally, no clear relationship was observed between pellet expansion and water stability across diets in our study. It is likely that factors other than pellet expansion alone contributed to its water stability. For example, the compositional characteristics of the fungal biomass may enhance pellet matrix cohesion and resistance to disintegration in water. From a practical point of view, a higher water stability index is generally advantageous as it ensures that the feed remains intact, particularly in aquatic environments, until consumed by the fish [22, 34]. It is also not beneficial to have a reduced pellet durability as it can lead to greater losses during transportation due to pellet breakage and the formation of fines [35]. Future work should focus on optimising processing conditions for microbial ingredients to enhance pellet durability without compromising water stability, ultimately improving pellet quality and feed efficiency.

In addition to pellet physical quality, growth performance provides a critical measure of the biological suitability of microbial ingredients in aquafeeds. Our results showed that *P. variotii* can partially replace fish meal and soy protein concentrate as an alternative dietary protein source in diets for juvenile rainbow trout without adversely affecting growth performance at inclusion levels of up to 20% using the current diet formulation and feed manufacturing. For instance, fish fed the control diet and those fed with *P. variotii*‐based diets containing up to 20% inclusion exhibited similar growth responses, as reported by similar final body weights and weight gain percentages over the 9‐week feeding period, although the diets were not isonitrogenous or isoenergetic, and extrusion conditions differed among treatments.

An interesting observation in this study was that increasing the inclusion level of *P. variotii* to 30% did not result in additional growth benefits and instead caused a significant reduction in growth compared with fish fed the control diet. Moreover, this reduction coincided with a significant decrease in feed intake. Under the present diet formulation and processing conditions, this close correlation indicates that the decreased feed intake could be one of the main contributing factors to the decreased growth observed in the group fed the *D*_30_ diet. The decreased feed intake in the *D*_30_ group may be due to differences in technical feed quality and taste as well as the presence of cell wall components or metabolites that could influence feed intake or gastrointestinal health. Additionally, the reduction in growth and the lower feed utilisation of fish fed the 30% *P. variotii* diet could be a result of the lower lipid and energy content and DP/DE ratio of these diets [36, 37].

Taken together, the growth reduction observed in our study suggests that beyond a certain inclusion level, the ability of fish to efficiently utilise the microbial protein source may be exceeded, possibly leading to nutritional or physiological constraints that compromise the growth efficiency. Our findings are in close agreement with those of Hooft et al. [13], who reported that replacing up to 20% of dietary protein with *P. variotii* in isonitrogenous and isocaloric diets did not negatively affect growth performance in Atlantic salmon during a 9‐week feeding trial. Conversely, Dahlberg [38] observed a significant reduction in growth in rainbow trout at inclusion levels of 20% and 30% after a shorter (44 days) feeding period. Another recent study evaluating mushroom‐derived ingredients from *Agaricus bisporus*, *Lentinula edodes* and *Pleurotus ostreatus* in rainbow trout reported no negative effects on growth performance, feed efficiency, somatic indices or digestive enzyme activity, despite the relatively high inclusion level used in the digestibility trial, where mushroom meals were incorporated at a 30:70 ratio of test ingredient to reference diet. However, subtle physiological alterations were observed, including increased hepatic carbohydrate accumulation and vacuolisation in fish fed *L. edodes*, suggesting that metabolic adjustments may occur even when growth performance remains unaffected [39]. These highlight potential species‐specific responses or differences arising from diet formulation, processing conditions, or experimental duration. The quadratic response observed for weight gain, characterised by an improvement up to 20% inclusion, followed by a marked decline at 30%, provides clear evidence of a threshold response, beyond which the benefits of microbial protein substitution decrease. Further studies exploring inclusion levels between 20% and 30% are warranted to more precisely define the upper limit for optimal growth and to better elucidate the factors constraining the performance at higher inclusion levels.

Further insight into the physiological responses to increasing dietary inclusion of *P. variotii* was provided by the VSI and HSI. The HSI and VSI values for rainbow trout recorded in our current study agree with those reported by Hossain et al. [40], with VSI (10.34–10.94) and HSI (1.13–1.24) falling within the expected ranges for juvenile rainbow trout. This suggests that the test diets did not induce abnormal organ development. A clear linear trend in VSI was observed with increasing *P. variotii* inclusion levels. An increased VSI is an indication of increased visceral mass, which may be associated with altered metabolic processing of excess dietary protein and energy partitioning and/or enlargement of visceral organs [41, 42]. In our study, the significant linear increase in HSI with increasing dietary *P. variotii* levels supports our assumption that the increase in VSI was at least in part associated with increased fat deposition in the liver and surrounding visceral tissues. From a production perspective, a lower VSI is more desirable as it indicates that fish have a higher proportion of edible flesh relative to viscera. Thus, while moderate inclusion levels of *P. variotii* (up to 20%) did not adversely affect growth or somatic indices, a higher inclusion level of 30% may negatively impact the carcass yield and overall production efficiency. This emphasises the need to optimise inclusion levels when incorporating microbial protein ingredients into rainbow trout diets.

Research on the application of *P. variotii* as a feed ingredient in salmonid aquafeeds remains limited. However, evidence from monogastric terrestrial animal studies indicates that *P. variotii* can serve as a digestible and nutritionally effective protein source. For example, Näsi [11] reported that replacing soybean meal with *P. variotii* did not significantly affect the digestibility of dry matter, crude protein or crude fat in pigs. Likewise, Näsi [12] found that partial substitution of soybean and fishmeal with *P. variotii* in poultry diets did not adversely affect the feed conversion ratio, although higher inclusion levels significantly increased feed intake. Additionally, replacing up to 15% of conventional feed ingredients with *P. variotii* in pig diets did not compromise the growth performance compared with control‐fed animals [43]. Consistent with these findings, our study demonstrated that the inclusion of *P. variotii* at 5%–20% of the diet did not adversely affect nutrient digestibility or nutrient retention, which is consistent with the comparable growth performance observed at these inclusion levels. These findings suggest that *P. variotii* protein was effectively digested and utilised by rainbow trout when included at 5%–20%. In contrast, the reduced nutrient digestibility and retention observed at the higher inclusion level of 30% coincided with decreased feed intake and reduced growth performance. This may indicate that limitations at high inclusion levels may not be the result of digestibility constraints of *P. variotii* protein but rather from feed intake‐related factors, including lower DP/DE ratio and/or possibly metabolic constraints associated with excessive microbial ingredient inclusion. Similar intake‐related responses at higher inclusion levels have also been reported in terrestrial animals [44], further supporting the existence of an optimal inclusion threshold.

The linear increase in ash ADC with increasing inclusion levels, especially in the diets containing 20% and 30% *P. variotii*, indicates that the mineral availability in the test ingredients may be higher. No differences in the ADC of protein or dry matter of the diet were found in the present study. In contrast, Gaudhaman et al. [45] reported that the crude protein digestibility of a diet with 30% substitution of *P. variotii* had dry matter and crude protein ADCs of 76% and 89.5%, respectively. These values were slightly lower than what was observed in this study, which could be due to the differences in dietary formulation between the two studies. Hooft et al. [13] observed dietary dry matter ADCs ranging from 74.2% to 76.7% at inclusion levels of 0%–20% in Atlantic salmon. At the same inclusion levels, crude protein digestibility ranged from 86.8% to 89.8%, which was lower than the values observed in the present experiment. Additionally, Hooft et al. [13] reported that the dietary crude protein ADC at the 20% inclusion level was significantly lower than at the lower inclusion levels, as well as Dahlberg [38] observed that the ADC of DM ranged from 77.2% to 81.4% and CP ranged from 91.2% to 92.8%, which was similar to what was observed in this study.

The whole‐body compositions of ash, crude protein, crude fat, gross energy, and minerals (Ca, K, Mg, Na, P and S) did not differ among the different inclusion levels tested. This can be considered a positive outcome as the composition of the fish did not differ from the control diet. This, along with retention data, can provide important information on the nutritional suitability of the feed ingredient. Hooft et al. [13] suggested that increasing the inclusion levels of microbial ingredients may enhance nitrogen retention, partly due to their high nucleic acid content. This observation aligns with findings from other studies that have also reported improved nitrogen retention in response to microbial ingredient supplementation [46–48]. These effects may be explained by the fact that crude protein estimates include non‐protein nitrogen sources, such as nucleic acids [49], and microbial ingredients are particularly rich in these compounds [50]. Nucleotides, the building blocks of nucleic acids, can be synthesised either via the de novo pathway, using amino acid precursors, or via the salvage pathway, which recycles dietary nucleotides. The high nucleotide content of microbial ingredients may have reduced the need for de novo synthesis, thereby sparing amino acids for other metabolic functions and improving the nitrogen balance. The energy retention followed a similar trend to nitrogen retention, with lower energy retention in *D*_30_ inclusion, and a significant quadratic relationship was observed. Lower nitrogen retention may indicate poorer digestibility of energy, amino acid composition/levels, and/or gut health [51]. The crude fat retention was lowest at *D*_30_ and highest at *D*_20_, again indicating a strong quadratic relationship with increasing inclusion levels. The differences in crude fat retention could be due to differences in lipid composition as rainbow trout digests polyunsaturated lipids better than saturated lipids [52]. However, further studies on crude fat digestibility will give us more information. Phosphorus retention levels were higher in the control diet than at *D*_30_, showing a significant quadratic trend. Unlike plant ingredients, microbial ingredients do not contain phytate P, which is not readily available to monogastric animals [53]. Hence, the reduction in phosphorus retention at the highest inclusion level could be due to the overall reduced digestibility.

Beyond nutrient retention and growth metrics, the physiological suitability of an ingredient is fundamentally linked to intestinal health and function [54]. To this end, we evaluated the effects of graded *P. variotii* inclusion on gut morphology and barrier function using histology and the Ussing chamber technique. The results revealed that the 30% inclusion diet elicited an increase in villus length, which could indicate a compensatory or stimulatory response within the intestinal mucosa. This aligns with the findings of Hooft et al. [13] and Mensah et al. [14] in Atlantic salmon for a similar microbial ingredient. A plausible explanation may be the higher nucleotide content in the microbial ingredients as dietary nucleotides have been shown to improve intestinal epithelial cell proliferation in mammalian models [55, 56]. Furthermore, bioactive nutrients such as sodium butyrate and phenylalanine are known to promote villus elongation, either by being directly absorbed by epithelial cells or by stimulating the release of growth factors [57, 58]. While these mechanisms were not assessed in the current study, they present promising avenues for future research to understand the exact reason for the increased villi length at higher inclusion levels.

Goblet cells secrete mucus, which forms a protective barrier along the intestinal lining [59]. The lack of significant differences in the goblet cell area across dietary groups, coupled with no reduction in villi length, suggests that diets containing up to 30% *P. variotii* did not induce intestinal inflammation. Functional assessments using the Ussing chamber provided further insights into regional intestinal responses. In the proximal intestine, the primary site of nutrient digestion and absorption, TER and SCC did not differ significantly among groups. This suggests that paracellular permeability and ion transport remain stable. However, TEP was significantly lower in the *D*_30_ group, which could result from reduced active ion transport across the membrane and/or increased paracellular transport. Our results also showed a significant negative linear correlation between *P. variotii* inclusion levels and TEP. This could indicate that *P. variotii* or some of its components interact with the intestinal epithelia’s ability to maintain an electric potential. In contrast, the distal intestine of fish fed the *D*_20_ diet exhibited significantly higher TER than that of the control, which could indicate decreased paracellular mobility and improved barrier function. No significant differences were observed in the SCC or TEP in this segment, implying that active transport processes remain unaffected. The proximal intestine is the primary site of digestion and absorption [60]. Hence, an impaired proximal intestine could potentially affect the digestibility of the diet and/or growth of the fish in the long term.

In summary, dietary inclusion of *P. variotii* modulates growth performance, feed physical quality and intestinal responses in rainbow trout in a dose‐dependent manner. Under the present diet formulation and processing conditions, inclusion levels up to 20% were well tolerated, maintaining growth performance, nutrient digestibility, intestinal integrity and barrier function while also improving feed water stability. In contrast, a further increase to 30% inclusion did not confer additional growth benefits and was associated with reduced feed intake, altered feed physical properties and indications of compromised intestinal function despite some adaptive morphological responses. Taken together, these results indicate that *P. variotii* represents a promising microbial protein source for the partial replacement of conventional ingredients in trout diets when included at moderate levels. Future studies should focus on refining diet formulation by identifying optimal inclusion levels of *P. variotii* and processing strategies, conducting longer‐term growth trials and evaluating the environmental sustainability and life cycle impacts of *P. variotii* production relative to conventional protein sources to support its potential commercial application.

## Author Contributions

**Ashwath Gaudhaman**: conceptualisation, formal analysis, investigation, methodology, visualisation, writing – original draft, writing – review and editing. **Aleksandar Vidakovic**: conceptualisation, investigation, writing – review and editing, supervision. **Niklas Warwas and Darragh Doyle**: investigation, methodology, writing – review and editing. **Byron Morales-Lange**: investigation, supervision, writing – review and editing. **Margareth Øverland**: investigation, supervision, writing – review and editing, funding acquisition. **Torbjörn Lundh**: conceptualisation, investigation, supervision, writing – review and editing, funding acquisition. **Kartik Baruah**: conceptualisation, investigation, supervision, writing – review and editing, funding acquisition, project administration.

## Funding

This study was supported by the NORDICFEED project ‘*Biokonvertering av bioresurser’* (Project Number 24931000) funded by FORMAS and NORDFORSK and the ForestFeed project ‘*En nordisk blågrön värdekedja från skog till fiskfilé’* (Project Number 2023‐00132) funded by VINNOVA.

## Conflicts of Interest

The authors declare no conflicts of interest.

## Data Availability

The data that support the findings of this study are available upon request from the corresponding author.
